# P2X7 Receptor at the Crossroads of T Cell Fate

**DOI:** 10.3390/ijms21144937

**Published:** 2020-07-13

**Authors:** Elizabeth Rivas-Yáñez, Carlos Barrera-Avalos, Brian Parra-Tello, Pedro Briceño, Mariana V. Rosemblatt, Juan Saavedra-Almarza, Mario Rosemblatt, Claudio Acuña-Castillo, María Rosa Bono, Daniela Sauma

**Affiliations:** 1 Departamento de Biología, Facultad de Ciencias, Universidad de Chile, Santiago 7800003, Chile; elizabeth.ecry@gmail.com (E.R.-Y.); brian.parrat@gmail.com (B.P.-T.); pedro.bcatalan@gmail.com (P.B.); mariana.rosemblatt.b@gmail.com (M.V.R.); juan.saavedra.a24@gmail.com (J.S.-A.); mrosemblatt@cienciavida.org (M.R.); 2 Departamento de Biología, Facultad de Química y Biología, Universidad de Santiago de Chile, Santiago 9160000, Chile; carlos.barrera@usach.cl; 3 Facultad de Medicina y Ciencia, Universidad San Sebastián, Santiago 7510157, Chile; 4 Fundación Ciencia & Vida, Santiago 7780272, Chile; 5 Centro de Biotecnología Acuícola, Universidad de Santiago de Chile, Santiago 9160000, Chile

**Keywords:** P2X7 receptor, ectonucleotidases, T cell

## Abstract

The P2X7 receptor is a ligand-gated, cation-selective channel whose main physiological ligand is ATP. P2X7 receptor activation may also be triggered by ARTC2.2-dependent ADP ribosylation in the presence of extracellular NAD. Upon activation, this receptor induces several responses, including the influx of calcium and sodium ions, phosphatidylserine externalization, the formation of a non-selective membrane pore, and ultimately cell death. P2X7 receptor activation depends on the availability of extracellular nucleotides, whose concentrations are regulated by the action of extracellular nucleotidases such as CD39 and CD38. The P2X7 receptor has been extensively studied in the context of the immune response, and it has been reported to be involved in inflammasome activation, cytokine production, and the migration of different innate immune cells in response to ATP. In adaptive immune responses, the P2X7 receptor has been linked to T cell activation, differentiation, and apoptosis induction. In this review, we will discuss the evidence of the role of the P2X7 receptor on T cell differentiation and in the control of T cell responses in inflammatory conditions.

## 1. Introduction

The purinergic signaling pathway is a highly conserved signaling pathway regulating several critical aspects of the immune response. Purinergic signaling is mediated by purine nucleotides and nucleosides such as adenosine triphosphate (ATP), nicotinamide adenine dinucleotide (NAD^+^), adenosine, and different receptors that are widely expressed on immune cells [1,2]. In homeostatic conditions, extracellular ATP (eATP) is found at nanomolar concentrations; however, under conditions of stress or cellular damage, ATP is released by necrotic or apoptotic cells, accumulating at micromolar concentrations. Since ATP is released during tissue damage, it is considered a potent “danger signal” or damage-associated molecular pattern (DAMP) [3] mediating the recruitment of effector immune cells [4] and promoting the removal of damaged cells by phagocytes in the inflamed tissue [5]. ATP may also be released during T cell activation through pannexin-1 channels, participating in a positive feedback loop that promotes T cell activation [6].

Extracellular ATP is rapidly hydrolyzed by nucleotidases located at the plasma membrane of numerous cells, generating ADP, adenosine monophosphate (AMP), and adenosine [4]. These reactions are catalyzed by members of four families of ectonucleotidases that include the ectonucleoside triphosphate diphosphohydrolase (eNTPD) family, the ectonucleotide pyrophosphatase/phosphodiesterase (eNPP) family, the alkaline phosphatase family, and CD73 (ecto-5ʹ-nucleotidase). CD39 (NTPDase-1) and CD73 constitute the main ectonucleotidases present on immune cells and have been considered as pivotal in regulating immunity and inflammation [7,8]. CD39 catalyzes the conversion of ATP to AMP [9], and CD73 catalyzes the dephosphorylation of AMP to adenosine [8,10]. Adenosine may act on P1 receptors on T cells (such as A2AR) and reduce T cell activation [11]. By catalyzing the hydrolysis of ATP to adenosine, a well-known immunosuppressive molecule, these ectonucleotidases may be considered as "immunological switches", tilting the balance from inflammation to immunosuppression [12].

Purinergic receptors are divided into three major families based on their structural properties: the P2X receptors, the P2Y receptors, and the P1 receptors. P1 receptors are G-protein coupled receptors that are activated by adenosine. P2YRs are also G protein-coupled receptors that are activated by different nucleotides such as ATP, ADP, uridine triphosphate (UTP), uridine diphosphate (UDP), and UDP-glucose [13]. P2X receptors are ligand-gated, cation-selective channels permeable to Na^+^, K^+^, or Ca^+2^. Its main physiological ligand is ATP, but its function can be modulated by non-nucleotide ligands such as polymyxin B, LL-37, and serum amyloid A [14,15,16]. P2XRs can be subclassified by their affinity to ATP, into P2X1R, P2X2R, P2X3R, and P2X4R, which are triggered by low concentrations of ATP, whereas P2X7 receptor (P2X7R) has a remarkably high trigger threshold at millimolar concentrations of ATP (>100 uM) [11].

The P2X7R is unique among P2X receptors since, as it presents low affinities for ATP (EC_50_ >100 μm), it may be activated during inflammation. Moreover, this receptor triggers different responses depending on ATP concentrations; in low ATP concentrations, P2X7R can function as an ion channel, whereas in higher ATP concentrations or after sustained ATP signaling, P2X7R can form large-conductance pores, leading to apoptosis [11]. The P2X7R is widely distributed throughout the mammalian body and is expressed in cells of the hematopoietic lineage, including macrophages, dendritic cells, monocytes, eosinophils, mast cells, T and B lymphocytes, and neutrophils [17,18].

**Figure 1:**
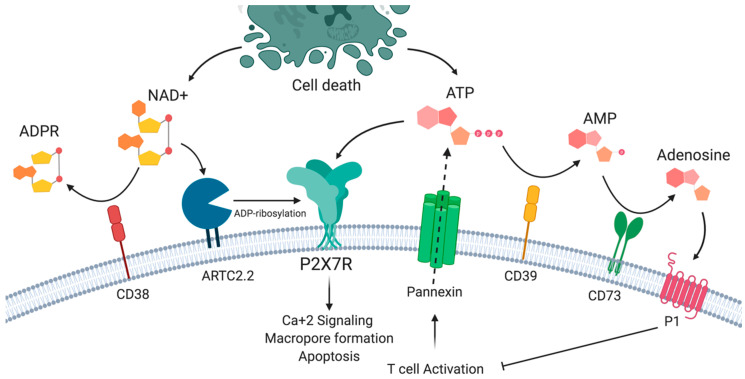
**The P2X7 receptor and purinergic signaling**. P2X7R may be activated by ATP and by ADP-ribosylation in the presence of nicotinamide adenine dinucleotide (NAD^+^). P2X7R activation results in the influx of sodium and calcium ions, phosphatidyl serine exposure, and macropore formation, leading to cell death. ATP and NAD^+^ availability is a result of the balance between the release by activated and dying cells and the action of extracellular nucleotidases such as CD39 and CD38.

In addition to ATP, P2X7R is activated by nicotinamide adenine dinucleotide (NAD^+^), which may also be released from damaged cells or intact T cells following T cell receptor activation [19,20,21]. This ATP-independent mechanism involves the action of ARTC2.2 ecto-ADP-ribosyltransferase expressed in the plasma membrane of murine lymphocytes. In the presence of low concentrations of NAD^+^ substrate, ARTC2.2 can catalyze ADP-ribosylation of the P2X7R in Arg125, which is close to ATP binding in the extracellular loop. ADP-ribosylation induces receptor activation, which is reflected in Ca^+2^, Na^+^ input, the exposure of phosphatidylserine (PS), DNA fragmentation, propidium iodide uptake, the breakdown of mitochondrial membrane potential, cells shrinkage and finally, NAD^+^-induced cell death (NICD) [22,23]. As in the case of ATP, NAD^+^ may be degraded by the CD38 ecto-NAD^+^ glycohydrolase mainly into ADP ribose (ADPR), cyclic ADP ribose (cADPR), and nicotinic acid adenine dinucleotide phosphate (NAADP) [24,25] (Figure 1). However, the relevance of this process of NICD in human T cells is unclear, since the gene encoding ARTC2 is not transcriptionally active, as a consequence of premature stop codons in the orthologous pseudogene [26].

We and others have postulated that CD39 and CD38 expression may provide T cells with the capacity to reduce the extracellular concentrations of ATP and NAD^+^, thus functioning as regulators of P2X7R-induced responses [12,27]. Interestingly, it has been proposed that CD39 and CD38 ectonucleotidases may also impact the extracellular levels of NAD^+^ and ATP, affecting other cells in trans. For instance, it has been shown that in murine splenocytes, B cells express the highest levels of CD38, and the depletion of B cells increases ADP ribosylation of several proteins on T cells, indicating that CD38 exerts this control not only in cis on the same cell surface as ARTC2.2 but also in trans on the surface of other cells [28]. Another report has shown that Langerhans cells may have a protective role in dermatitis by reducing the levels of extracellular nucleotides released by injured keratinocytes in a murine model of dermatitis. In this study, the authors demonstrate that extracellular nucleotides released during injury constitute inflammatory mediators in the skin by acting on keratinocytes and other cells. As Langerhans cells were demonstrated to express CD39 amongst the epidermal compartments, these results suggest that CD39 expressed by Langerhans cells (and other cells) may hydrolyze ATP, thus reducing extracellular nucleotide levels following injury with skin irritant chemicals [29].

During inflammation, several danger signals can be released into the extracellular space, including not only ATP but also Ca^+2^, both of which can regulate the response of different cells of the immune system [30,31]. It has been described that several divalent cations, and Ca^+2^ in particular, can alter the affinity of ATP binding to the P2X7 receptor, acting as allosteric inhibitors [32,33,34]. Thus, it is plausible that P2X7R activity might not only be fine-tuned by the availability of ATP and the activity of ectonucleotidases, but also by the levels of extracellular calcium in physiological conditions and during the inflammatory response.

## 2. P2X7 Receptor Structure and Functions

The P2X7R is encoded by the *p2rx7* gene and is the largest receptor within the P2X family of receptors, with a length of 595 amino acids for human, rat, and mouse receptors [35,36,37,38,39]. Recently, the crystal structure of mammalian P2X7R in complex with different antagonists has been reported [40]. Its monomeric structure has two intracellular domains, one C-terminal and one N-terminal, as well as two hydrophobic segments (transmembrane domains) separated by a long extracellular ATP-binding domain [39]. When activated by extracellular ATP, the P2X7R responds either as a non-selective cation channel or by mediating the activation of a series of intracellular signaling pathways [41]. It has been proposed that this dual function can be explained by differential distribution along the plasmatic membrane [42]. Localization of this receptor in lipid raft regions would enable it to maintain its monomeric conformation and activate intracellular signaling pathways [42]. In contrast, distribution along non-lipid raft regions would allow the P2X7R to form homotrimers in the presence of its agonist and function as an ion channel, allowing the entry of Ca^+2^.

In response to high concentrations or prolonged exposure to ATP, P2X7R generates macropores, induces membrane blebbing [43], and ultimately induces cell death [39,44]. The P2X7R macropore is characterized by a conductance with an upper limit of approximately 900 Da [39] and is permeable to exogenously applied fluorescent dyes, e.g., propidium iodide, YO-PRO1, and ethidium bromide [37,45]. The molecules and mechanisms involved in macropore formation are still under debate. 

It has been proposed that the macropore formation requires molecules extrinsic to P2X7R, such as connexin-43 and pannexin-1 channels [46,47,48]. In this line, it has been reported that the use of pannexin-1 antagonists and anti-pannexin-1 RNAi causes a decrease in P2X7R pore formation [48]. In addition, the co-expression of P2X7R with pannexin in oocytes provided evidence that pannexin channels may be the pore-forming units activated by the ATP stimulation of P2X7R [47]. However, connexin and pannexin-1 antagonists and pannexin-1 siRNA failed to inhibit ATP-induced pore formation following P2X7R activation in murine macrophages [49].

On the other hand, recent evidence supports an intrinsic role of P2X7R in the macropore formation, since it has been demonstrated that liposomes reconstituted with P2X7R show YO-PRO1 uptake in a dose-dependent manner [50]. In addition, truncations or mutations of P2X7R subunits abrogate the uptake of fluorescent dyes, accompanied by decreased cation fluxes [51,52]. Moreover, macrophages from P2X7R knockout mice do not display cationic fluorescent dye translocation in response to ATP [53]. All this evidence suggests that macropore formation is probably intrinsic to P2X7R, but the role of accessory molecules cannot be excluded [13].

In addition to its function as an ionotropic receptor and macropore formation, P2X7R has been linked to the activation of numerous signaling pathways that include phospholipases A2, D, and C, neutral sphingomyelinase, MAP kinases (extracellular signal-regulated kinase 1/2; ERK 1/2) [54,55], p38 [56], activation of transcription factors such as the cyclic AMP (cAMP) response element (CREB) [57] and metalloproteases activation [58].

The functionality of P2X7R can be affected by various factors within cells of the immune system. An essential factor to consider is the alternative splicing of the P2X7 transcript [59,60]. In mice, P2X7a is the common mRNA, and four alternative splice variants have been described; P2X7b, c, d, and k [61], where P2X7k is the variant predominantly expressed in T cells [60,62]. The P2X7k isoform is eight times more sensitive to P2X7R agonists than P2X7a [59], and only P2X7k is sensitive to ADP-ribosylation [60]. Another factor affecting the P2X7R function is allelic variants of the *p2rx7* gene within different murine strains [63]. C57BL/6 mice present a natural P451L allelic mutation that only impairs the functionality of the P2X7a isoform [62]. Finally, an additional factor affecting P2X7R activity among cell types is the surface expression levels of P2X7R. It has been described that in C57BL/6 mice, CD4^+^ CD25^+^ T cells express higher levels of P2X7R on their surface than CD4^+^ T cells and CD8^+^ T cells (being lower in CD8^+^ T cells), which correlates with their higher sensitivity to ATP and NAD^+^ [64,65]. Interestingly, in BALB/c mice, P2X7R expression on CD4^+^ and CD8^+^ T cells is higher than in C57BL/6 mice, and so is their sensitivity to ATP and death induced by this molecule [64]. This difference in P2X7R expression may explain why T cells from BALB/c mice are more sensitive to nucleotide-induced cell death than T cells from C57BL/6 mice [63,66].

A human equivalent to P2X7k has not been described [59,62], but it has been demonstrated that human resting CD4^+^ and CD8^+^ T cells express the common transcript P2X7A and the P2X7B variant [67]. P2X7B is the predominant transcript in many tissues, including cells of the immune system [67]. P2X7B assembly lacks the pore function but retains channel activity; it is more efficient in supporting cell growth than P2X7A and can heteromerize with P2X7A to produce an enhanced response compared to the P2X7RA homotrimer [52,61,67,68,69]. In addition, some reports have suggested the involvement of P2X7RB in human diseases [70,71]. One of the most widely studied non-synonymous single nucleotide polymorphism (SNP) resides at nucleotide 1513, which changes a glutamic acid to an alanine acid at amino position 496 (Glu496Ala), generating the loss-of-function of P2X7R. This SNP has been linked to an increased susceptibility to infection by *Mycobacterium tuberculosis* [72] and lymphocytic leukemia [73]. It is also associated with defects in CD62L shedding [74] and cytokine production in human leukocytes [75] in response to ATP. All this evidence supports a key role of the P2X7 receptor in a functional immune response.

## 3. P2X7 Receptor on Dendritic Cells

Dendritic cells (DCs) are specialized antigen-presenting cells that efficiently activate naïve T cells. Dendritic cells are poised in epithelial surfaces that are in contact with pathogens. These cells express P2X7R and respond to ATP released during tissue damage in an ongoing immune response. Following pathogen recognition, DCs become activated and migrate to draining lymph nodes to present the antigens derived from the pathogens to T cells. In the lymph node, T cell fate is mainly influenced by cytokines secreted during T cell activation by DCs. It is well recognized that cytokines such as interleukin (IL)-12, IL-4, IL-6, IL-23, IL-1β, and transforming growth factor beta (TGF-β) trigger the polarization of CD4^+^ T cells to Th1, Th2, Th17, and Treg cells [76,77]. In the following section, we will describe the effects of ATP sensing on different physiological functions of DCs, which in turn, determine T cell differentiation.

### 3.1. Inflammasome Activation

Perhaps the most well-recognized effect of P2X7R activation induced by ATP on DCs is IL-1β production through the inflammasome assembly. The inflammasome consists of a large multiprotein complex composed of an NLR (nucleotide-binding oligomerization domain-like receptor; NOD-like receptor), the ASC adaptor protein, and procaspase 1. Its assembly leads to the activation of caspase 1, which results in the cleavage of pro-IL-1β and pro-IL-18 to generate the active pro-inflammatory cytokines. Numerous inflammasomes have been identified; however, the best characterized is the NLRP3 inflammasome [78,79,80]. The inflammasome activation and IL-1β secretion might have important consequences on T cell differentiation, as this cytokine has been reported as crucial and required for the priming of tumor-specific interferon-gamma (IFN-γ)-producing CD8^+^ T cells and has been shown to influence the growth and differentiation of T helper subsets such as Th17 cells [81,82].

A putative role of P2X7R on inflammasome activation in human DCs was first suggested in 2000. In this report, the authors showed that human DCs respond to ATP by increasing intracellular calcium and that LPS matured DCs secrete IL-1β and TNF-α upon incubation with ATP [83]. Later studies demonstrated P2X7R involvement in inflammasome activation in the context of tumor immunity. These reports suggested that upon ATP release, P2X7R is activated, inducing the NLRP3-dependent inflammasome assembly, leading to IL-1β production and the priming of IFN-γ producing T cells [81]. Accordingly, P2X7R-/- DCs produced less IL-1β when cultured with tumor cells than wild-type DCs [84]. On the other hand, the ex vivo supplementation of exogenous ATP on lipopolysaccharide-primed bone marrow-derived DCs increased the expression of P2X7R, NLRP3, ASC, and caspase-1, while this effect was attenuated by treatment with apyrase [85]. The mechanisms behind NLRP3-dependent inflammasome activation are still unknown, but it has been proposed that K+ efflux through the P2X7R-dependent pore and pannexin-1 hemichannels is required for this process. However, a role for intracellular calcium increase in this process cannot be discarded [68].

### 3.2. DC Maturation and Migration

Danger signals such as ATP can stimulate DC migration to the draining lymph node, where they can activate naïve lymphocytes and enhance the immune response [86]. In this line, in vitro studies have reported that ATP induces the expression of CCR7 and CXCR4, two chemokine receptors that are involved in the migration to lymph nodes and peripheral tissues, respectively [87,88].

It has also been demonstrated that signaling through the pannexin-1 channel and the P2X7R is responsible for the fast ATP-dependent migration of DCs. This signaling is required for the actin cytoskeleton reorganization associated with DC migration [89]. In agreement with reports that show that P2 receptor stimulation leads to an increase in cytosolic calcium [89,90], Saez and collaborators demonstrated that calcium flux was necessary for ATP-mediated fast DC migration. It has been reported that calcium/calmodulin-dependent protein kinase II (CaMKII), a major downstream effector of calcium, is involved in the regulation of actin cytoskeleton remodeling that directly affects DC migration [91].

Normal DC maturation involves an increase in the surface expression of co-stimulatory molecules such as CD80, CD86, CD40, and MHC molecules [17,70]. In this line, it has been reported that the exogenous addition of ATP (10 uM) induces CD80 and CD86 expression in bone marrow-derived dendritic cells, resulting in the efficient activation of CD4^+^ T cells. In addition, the in vivo depletion of ATP with apyrase reduced CD80 and CD86 expression in lymph node–CD11c+ cells in an acute graft-versus-host disease murine model [92]. Further evidence suggested that ATP’s effect on DC maturation may be mediated by the induction of nuclear factor kappa B (NF-kB) (p65) expression [93].

### 3.3. Cytokine Secretion and Polarization of T Helper Cells

#### 3.3.1. Th1 Cell Differentiation

Although there is evidence of a possible role for ATP on DCs reducing Th1 responses in vitro, there are few reports on the specific effects of P2X7R on DCs during Th1 differentiation. In vitro experiments have shown that DCs stimulated with micromolar concentrations of non-hydrolyzable ATP inhibit the production and secretion of IL-12 and TNF-α [94]. Accordingly, DCs pretreated with ATP impaired Th1 polarization when co-cultured with naive CD4^+^ T cells [94]. However, the role of P2X7R reducing IL-12 production by DCs is still under debate, since other studies have also indicated that the P2Y_11_ receptor might mediate this effect through cAMP signaling [95,96]. On the other hand, ATP may not only reduce Th1 cell differentiation but also decrease the interaction of DCs with T cells. Treating DCs with micromolar concentrations of ATP reduces their ability to attract Th1 and cytotoxic lymphocytes. Additionally, the supernatants obtained from ATP-treated DCs have demonstrated to decrease the migratory capacity of these lymphocytes [97].

#### 3.3.2. Th17 Cell Differentiation

The ATP/P2X7R axis has been described as a modulator in Th17 polarization in different tissues and models [98,99,100,101,102,103,104]. Studies in heart and pancreatic islet transplant models in mice showed that the use of a P2X7R antagonist (periodate-oxidized ATP, oATP) induced a decrease in the Th17 differentiation and had beneficial effects for transplantation [99,100].

The effect of P2X7R activation on Th17 cell differentiation has been studied in autoimmune diseases as well. Reports in lupus-prone mice, where P2X7R is upregulated compared to control mice, showed that the renal expression of NLRP3, ASC, and caspase 1p20 was enhanced and correlated with higher renal and serum levels of IL-1β [101]. In this work, the use of the P2X7R antagonist (brilliant blue G, BBG) or P2X7R siRNA treatment reduced the expression of these molecules and decreased the frequency of spleen Th17 cells and serum IL-17 levels [101]. This effect of P2X7R was also observed in a study of myasthenia gravis patients, where the culture of peripheral blood mononuclear cells from patients challenged with lipopolysaccharide showed an increase in the expression of IL-1 β, IL-6, and IL-17, which was reduced by blocking P2X7R (with BBG) [104]. Accordingly, P2X7R induced IL-1β, IL-6, and IL-17 expression in human visceral adipose explant cultures treated with a specific P2X7R agonist (BzATP), and it was inhibited by treatment with a specific P2X7R antagonist (KN-62) [103]. Although in all these studies, the direct participation of DCs in the process is not demonstrated, they support the hypothesis that the release of crucial cytokines such as IL-1β, IL-6, and IL-23 through P2X7R activation in DCs contributes significantly to Th17 cell differentiation.

Evidence of the direct effect of P2X7R activation in DCs driving Th17 polarization arises from the study of the Atarashi’s group [105]. In 2008, Atarashi et al. showed that stimulation with ATP analog (αβ-ATP) induces the expression of IL-6 and IL-23 in a lamina propria DC subset that expresses P2X7R [105]. Similarly, in a later study, the culture of migratory human skin DCs with the P2X7R agonist BzATP produces an increase in the expression of maturation markers such as CD86 and HLA-DR, in addition to inducing the expression of IL-1β, IL- 6, and IL-23 [70]. In this work, the induction of these cytokines was not observed when DCs were cultured in the presence of the P2X7R antagonist KN-62 [70]. This effect was also observed in a study based on the type II collagen-induced arthritis model on mice, where the culture of bone marrow-derived DCs in the presence of type II collagen-induced the expression of P2X7R and cytokines such as IL-1β, IL-6, IL23p19, and TGF-β1. In contrast, this cytokine expression was strongly inhibited when the DCs were pretreated with a P2X7R antagonist (suramin and A-438079) [106]. In all these studies, the presence of IL-6, IL-1β, or IL-23-producing cells, under P2X7R activation conditions, increased Th17 responses and were inhibited with P2X7R antagonists.

Additionally, it has recently been described that the ATP/P2X7R axis DCs activates the NLRP3 inflammasome, promoting the differentiation to Th17 cells in an ovalbumin-induced murine asthmatic model [85]. The role of the inflammasome in this context was demonstrated by the use of caspase-1 and NLRP3 inhibitors in DCs, both of which reduced Th17 cell differentiation [85].

**Figure 2:**
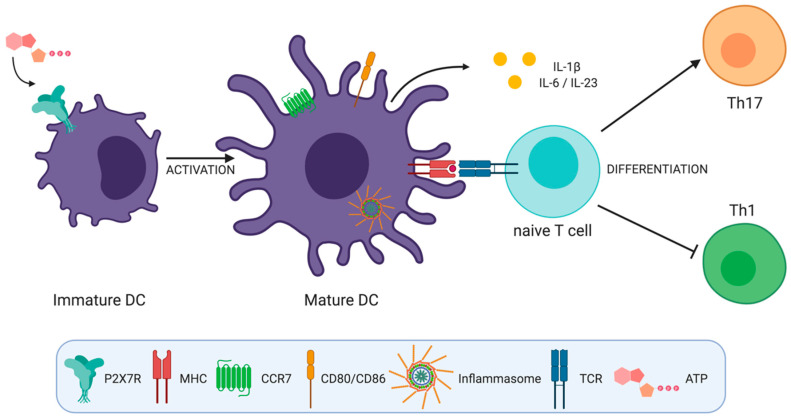
**ATP induced activation of P2X7 receptor on dendritic cells (DCs) has several effects that impact T helper cell differentiation**. P2X7R activation by ATP (a) induces inflammasome activation, (b) favors DC migration by inducing calcium flux and chemokine receptor expression on DCs, (c) induces DC activation and the expression of CD80 and CD86 co-stimulatory molecules and (d) induces interleukin (IL)-1β, IL-6, and IL-23 production by DCs favoring Th17 over Th1 cell responses.

All these works suggest that the activation of P2X7R in DCs plays a role inducing Th17 cell responses by generating a cytokine microenvironment that favors the differentiation of these cells (Figure 2).

## 4. P2X7 Receptor on T Cells

### 4.1. T Cell Activation

Purinergic signaling at the immune synapse plays a vital role in the amplification of T cell receptor (TCR) signaling. Following antigenic recognition, signal transduction from the TCR and co-stimulatory molecules induces the rapid release of micromolar concentrations of ATP through pannexin channels by T cells [11,107,108]. ATP released in the immunological synapse acts in an autocrine fashion to activate P2X receptors, including P2X1, P2X4, and P2X7 receptors [6]. P2X activation results in a Ca^+2^ influx, IL-2 production, and proliferation by the activation of NFAT (activated T cell nuclear factor) along with an increased expression of the *p2rx7* gene [11,108]. Following activation, P2X1 and P2X4 receptors are rapidly recruited to the immunological synapse, whereas the P2X7R remains uniformly distributed at the cell surface where it may sense environmental ATP [107].

The role of P2X7R on calcium influx and downstream signaling required for T cell activation was reported on human CD4^+^ T cells and Jurkat cells by the group of Junger. In this study, the authors demonstrated that P2X7R inhibition through different strategies such as the removal of extracellular ATP by apyrase, inhibition of ATP release with gadolinium chloride, pharmacological P2X7R blockade, or siRNA silencing of P2X7R all result in calcium entry blockade and the inhibition of T cell activation [108]. In addition, it has been reported that P2X7R knockout mice present an altered leukocyte function and inflammatory response [109]. This data suggests that in combination with the signals generated at the immune synapse, P2X7R activation by ATP is necessary for the complete activation and proliferation of T lymphocytes.

### 4.2. T Cell Migration and Motility

L-selectin (CD62L) is fundamental for T cell migration, as it allows the entry of lymphocytes to secondary lymphoid tissues via high endothelial venules [110]. L-selectin present on leucocytes binds to peripheral node addressins (PNAd) present on endothelial cells, allowing transendothelial migration and entry into secondary lymphoid organs [111]. Following T cell activation and differentiation, T cells must reduce CD62L expression in order to egress from the lymph node [112]. It has been suggested that P2X7R may have a role in T cell egress from the lymph node, since ATP induces CD62L shedding from the plasma membrane of lymphocytes as a result of metalloproteases ADAM10 and ADAM17 activation [58]. Later, it was demonstrated that P2X7R activation by ATP increases mitochondrial superoxide generation in human naïve T cells, triggering CD62L shedding [113]. New reports have shown that recently activated naïve T cells, which express CD69, present a decreased shedding of CD62L compared to naive T cells. These results suggest that the autocrine activation of P2X7R could amplify the transient retention of recently activated T cells in the lymph node caused by CD69-mediated inhibition of S1RP1, favoring a full differentiation of activated T cells into effector cells [114].

In addition to its effects on T cell migration, new evidence has demonstrated that P2XR activation during T cell activation in the lymph node may have an impact on T cell motility. This report demonstrated that ATP released by a stimulated T cell acts in a paracrine fashion to induce calcium waves in the neighboring T cells through P2X4/P2X7 activation. This calcium wave reduces T cells’ motility, allowing the stability of DC–T cell conjugates [115].

### 4.3. Differential Sensitivity to ATP and NAD^+^ Induced Cell Death

Intracellular ATP and NAD nucleotides have been considered essential energy sources. However, during inflammatory processes and tissue injury, both nucleotides are released into the extracellular space [20,21] where they can trigger P2X7R activation, which results in the induction of apoptosis or necrosis depending on the incubation time, dose, and cell type [116].

Extracellular NAD^+^ is considered a substrate for the post-transcriptional modification of proteins such as phosphorylation and the ADP-ribosylation. Importantly, NAD^+^ serves as a substrate for ARTC2.2, which catalyzes the ADP-ribosylation of the P2X7R and other cell surface proteins on T cells. ADP ribosylation of P2X7R in the presence of extracellular NAD^+^ (at low micromolar concentrations) induces sustained P2X7R activation, resulting in cell death by apoptosis [23]. This process is known as NAD^+^ induced cell death (NIDC).

Sustained P2X7R activation either by ATP or NAD-dependent ADP ribosylation [23,27,117] leads to T cell death by two independent pathways, one of which depends on the cellular contraction given by the formation of a non-selective pore and the second involving the phosphorylation of ERK1/2 [118].

Prolonged triggering of P2X7R results in the formation of non-selective membrane pores dependent on the cytoplasmic C-terminal domain of P2X7R [39,52]. Pore formation causes a flow of ions associated with a change in cell size and the transient cellular contraction required for phosphatidylserine translocation [119,120]. Additionally, it has been observed that the activation of P2X7R triggers CD62L shedding, loss of the mitochondrial membrane potential, DNA fragmentation, and also increases phospholipase D activity, resulting in cell death by apoptosis [23,121,122]. However, the group of Elliot has reported that the initial shrinkage observed after P2X7R activation is quickly followed by cell swelling in lymphocytes, which ultimately ends in cell lysis. This observation suggests that P2X7R activation may induce a novel cell death pathway that does not fit the canonical apoptotic nor necrotic pathway [119].

On the other hand, P2X7R induced cell lysis or necrosis has been observed independent of pore formation and calcium influx. This pathway was found to require signal-regulated protein kinase 1/2 (Erk1/2) phosphorylation [118,123]. Although in other cell types, the N-terminus of P2X7R is important for Erk1/2 activation [54], the mechanisms of how this signaling pathway works on T cells have not been clarified.

#### 4.3.1. Expansion of Primed T Cells in Lymph Nodes

The delicate regulation of the P2X7R and ARTC2.2 expression is one of the primary mechanisms that may explain the differential susceptibility of different cellular populations to ATP and NAD-induced cell death in lymph nodes. Adriouch and collaborators demonstrated that P2X7R and ARTC2.2 are decreased at early stages following the activation of T cells compared to naive T cells, suggesting that naive T cells and memory cells are susceptible to NICD mediated by P2X7R. They proposed that NAD^+^ released during early inflammation facilitates the expansion of primed T cells, through the ARTC2.2-driven death of resting cells [124].

**Figure 3:**
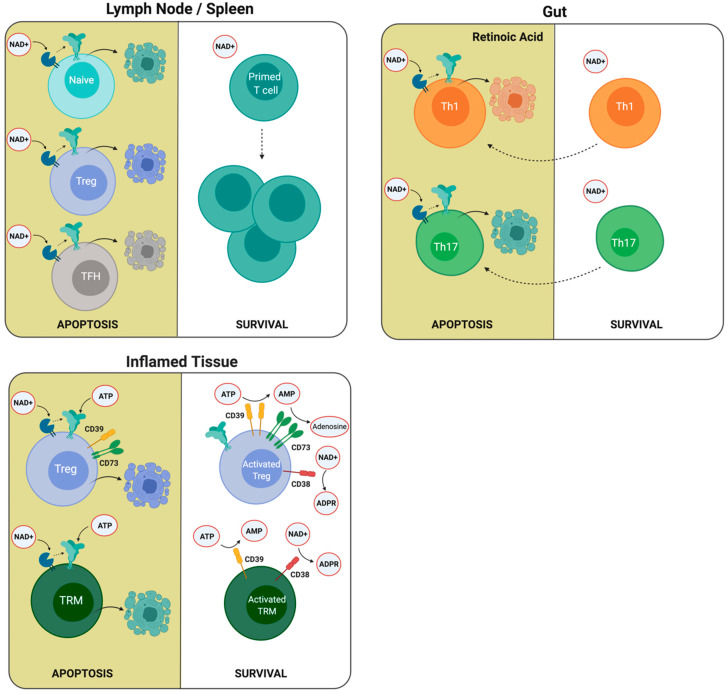
**T cells present different susceptibility to ATP and NAD^+^ induced cell death**. The differential expression of P2X7R and ARTC2.2, CD38, and CD39 enzymes may explain the susceptibility of different T cell populations to ATP and NAD^+^ induced cell death. Low P2X7R and ARTC2.2 expression in primed T cells compared to naïve cells, regulatory T cells (Tregs), and follicular helper T (T_FH_) cells in the lymph node may explain the expansion of primed T cells following antigenic stimulation. In the intestine, retinoic acid stimulates the expression of P2X7R, which increases the susceptibility to cell death. In inflamed tissues, Tregs and resident memory T cells (T_RM_) may upregulate the expression of CD38 and CD39 ectonucleotidases following antigenic stimulation, reducing NAD and ATP extracellular concentrations and rendering these cells less susceptible to ATP and NAD-induced cell death.

Besides being tightly regulated following activation, P2X7R expression is differentially expressed in different T cell populations. For instance, reports from the literature indicate that regulatory T cells (Tregs) exhibit the highest expression of P2X7R among T cells and are more susceptible to cell death by NAD^+^ or ATP in vitro [64,65,114,125,126]. This enhanced susceptibility of Tregs to NICD has also been explained as a mechanism that allows the expansion of primed T cells within the lymph nodes [124] (Figure 3). However, the role of ATP-induced cell death of Tregs in lymph nodes has not been sufficiently demonstrated, since extracellular ATP released in the inflammatory site is probably unable to reach the draining lymph nodes [127,128] because of its short half-life in body fluids due to widely distributed ecto-ATPases [129].

Other reports have demonstrated that follicular helper T (T_FH_) cells also express high levels of P2X7R, rendering this population very susceptible to NICD (Figure 3). The first evidence of a higher sensitivity of T_FH_ cells to NAD-induced cell death came from studies comparing T_FH_ and Th1 cells [130]. In this work, the authors compared genes related to the purine metabolic pathway and demonstrated that *nt5e* and *p2rx7* genes were expressed in T_FH_ cells. Interestingly, the *p2rx7* gene was highly expressed in T_FH_ cells located in Peyer’s patches, and the P2X7R controls their numbers, and consequently, the production of immunoglogulin A (IgA) against gut commensals [131]. Moreover, in 2017, Salles and collaborators demonstrated that the P2X7R controls the T_FH_ cell population in the context of *Plasmodium chabaudi* infection [132]. Through adoptive transfer experiments, the authors showed that the selective absence of the P2X7R receptor in CD4^+^ T cells is sufficient to increase T_FH_ cell frequency. An explanation of this phenomenon relies on the high susceptibility of T_FH_ cells to ATP-induced cell death, since as mentioned earlier, they express high levels of P2X7R and express relatively low levels of CD39 ectonucleotidase, which may be insufficient to degrade eATP rapidly and thus prevent ATP-induced cell death. In addition, it was demonstrated that P2X7R deletion enhanced the generation of pathogenic T_FH_ cells in a murine model of systemic lupus erythematosus. In this setting, P2X7R-mediated cell death was associated with the induction of pyroptosis as it was executed by gasdermin D [133]. Unexpectedly, other experiments to test memory responses using infection with lymphocytic choriomeningitis virus (LCMV) and preventing NICD using an ARTC2.2 blocking nanobody revealed that T_FH_ CD4^+^ T cells comprised a large proportion of the total antigen-specific memory CD4^+^ T cells in the spleen of infected mice [134].

#### 4.3.2. Regulation of P2X7R Expression in the Gut by Retinoic Acid

In contrast to peripheral lymph nodes, the intestine requires additional checkpoints to avoid excessive immune responses. A report shows that intestinal CD8^+^ T cells express higher levels of surface P2X7R as compared with splenic CD8^+^ T cells. Accordingly, intestinal CD8^+^ T cells are more susceptible to ATP (and NAD^+^) induced cell death in vitro. These data suggest that the P2X7R expression is not only regulated during T cell differentiation but also by tissue-derived specific factors [135]. Retinoic acid (RA) contributes to ATP and NAD-induced cell death since the incubation of splenic CD8^+^ T cells in the presence of RA upregulated the expression of P2X7R and ARTC2.2 [135].

In addition to its effect on CD8^+^ T cells, Hashimoto-Hill and collaborators have recently demonstrated that RA also induced P2X7R expression on effector T cells, and this allows the depletion of Th1 and Th17 cells in the intestine (Figure 3). In this report, it was demonstrated that the NAD-dependent ADP ribosylation of the P2X7R induces the contraction of intestinal Th1 and Th17 cell populations in the steady state and during active immune responses to bacterial pathogens. In vivo NAD treatment also depleted inflammatory effector T cells and suppressed tissue inflammation in the intestine [136].

#### 4.3.3. Survival of T Cells in Inflamed Tissues

As previously stated, Tregs may be rescued from ATP-induced cell death through the induction of the expression of CD39 and CD73, both of which cooperate in catalyzing the conversion of ATP into adenosine (Figure 3) [137,138]. Borsellino and collaborators showed a correlation of CD39 with Foxp3+ expression in Tregs and demonstrated that the catalytic activity of CD39 is dramatically increased following Treg activation. They propose that CD39 expression confer Tregs with the capacity to enter into an inflamed tissue with a high concentration of ATP to exert their suppressive function [139]. It has been demonstrated that Tregs upregulate CD38 expression following activation, and its expression correlates with their immunosuppressive activity [140,141,142] and thus may also escape NAD-induced cell death in lymph nodes [126] and possibly after entering inflamed tissues.

A fundamental characteristic of adaptative immunity is the generation of memory, which allows a faster and more effective response to reinfection. Three classic memory CD8^+^ T cell populations have been defined based on their homing abilities, cytokine production, and metabolic requirements: central memory, effector memory, and resident memory lymphocytes. Resident memory cells (T_RM_) constitute a subset of memory cells that reside in the tissues without recirculating [143]. Resident memory T cells (T_RM_) also may escape cell death by increasing the levels of ectonucleotidases upon T cell activation. It was demonstrated that following sterile injury, liver T_RM_ cells were highly susceptible to ATP and NAD^+^ induced cell death. However, the concomitant T cell receptor activation induced CD38 and CD39 nucleotidases and reduced T_RM_ susceptibility to P2X7R induced cell death [144] (Figure 3).

### 4.4. T Cell Differentiation and Function

#### 4.4.1. Control of CD8^+^ Memory T Cell Fitness

Extracellular ATP affects the differentiation and survival of activated T cells and influences the generation and functionality of memory CD8^+^ T cells [145]. In a murine model of acute lymphocytic choriomeningitis virus (LCMV) infection, the adoptive transfer of LCMV-specific WT and P2X7R KO CD8^+^ T cells showed that the generation of central memory lymphocytes was drastically decreased in P2X7R deficient T cells. The defect observed in memory differentiation in P2X7R-deficient T cells was already evident in the memory precursor stage, which showed altered metabolic functions, such as lower mitochondrial mass and reduced spare respiratory capacity, which are both critical for memory differentiation. In vivo and in vitro experiments demonstrated a significant role of P2X7R in the metabolic programming, survival, and function of memory T cells [145].

It has also been proposed that the AMP activated-protein kinase (AMPK)/mammalian target of rapamycin (mTOR) axis might be involved in the P2X7R induction of memory T cell differentiation and maintenance. AMPK promotes memory differentiation and can be regulated by ATP’s intracellular concentration [146]. Borges da Silva’s group showed that T cells defective in P2X7R show increased intracellular ATP concentrations, which is accompanied by a reduction in AMPK activity, since P2X7R can also regulate the outflow of ATP through pannexin-1 channels. AMPK can also be activated by Ca^2+^ influx, which is also elevated by P2X7R activation. Finally, AMPK activation inhibits mTOR activity, which ultimately favors memory differentiation [145].

A later study showed that a subpopulation of long-lived memory CD8^+^ T cells could be refractory to the effect of high extracellular ATP concentration, although they express similar levels of P2X7R as other CD8^+^ T cell population [147,148]. However, even if long-lived CD8^+^ memory T cells do not suffer the effects of high extracellular ATP, such as CD62L shedding, pore formation, and cell death, lower concentrations of this nucleotide might be beneficial to the long-term maintenance of memory cells. It has been proposed that the positive feedback loop between P2X7R and pannexin-1 channels in memory T cells maintains a constant efflux of intracellular ATP that could favor their long-term survival, perhaps through AMPK signaling [145,149]. Therefore, P2X7R is essential for the differentiation of long-lived memory T cells, both by modulating their metabolic fitness but also by regulating intracellular levels of ATP and Ca^2+^ that ultimately influence the activity of AMPK and impact memory T cell maintenance.

#### 4.4.2. Control of Regulatory T Cell Differentiation and Plasticity

Regulatory T cells are identified by the expression of a key transcription factor, known as forkhead box P3 (Foxp3), that is required for their development, maintenance, and function [150,151]. The principal role of Tregs include maintaining peripheral tolerance, preventing autoimmunity, and limiting chronic inflammatory diseases through a set of mechanisms involving cell–cell contact, the depletion of IL-2, and the release of soluble inhibitory factors [138].

As previously stated, the effect of ATP on T cell function is entirely dependent on its concentration. Trabanelli and collaborators showed that ATP at low physiological concentrations did not modulate either the proliferation or cell death of activated Tregs. Conversely, high ATP concentrations (1 mM) can “turn on” Treg cells, making them capable of exerting their immunosuppressive functions [152]. However, this effect depends on the level of activation and CD39/CD73 expression by Tregs. It has been demonstrated that high ATP concentrations, such as the levels following tissue injury together with the low catalytic activity of CD39 in non-activated Tregs, induce P2X7R activation and may affect the stability of Tregs. In this line, P2X7R activation in the presence of the pro-inflammatory cytokine IL-6 increases the extent of ATP synthesis and ERK phosphorylation in Treg cells through the autocrine activation of P2X7R. All this results in an impairment of the suppressive function and lineage stability of Tregs, inducing the differentiation to Th17 cells [125].

Another type of regulatory T cells is the Tr1 cells, which are characterized by the high production of IL-10 and the absence of Foxp3 [153]. Evidence for the purinergic signaling control on the differentiation of Tr1 cells was suggested by the group of Quintana [154]. In this report, P2X7R activation by ATP inhibited the differentiation of Tr1 cells, while the frequency of this population was increased in P2X7R-deficient mice. CD39 expression was demonstrated to be necessary for the production of IL-10 by Tr1 cells and allowing the depletion of ATP. A possible explanation for this phenomenon is that the reduction of ATP sensing allows aryl hydrocarbon receptor (AHR)/AHR nuclear translocator (ARNT) interaction that increases the production of IL-27 and favors Tr1 differentiation [155].

**Figure 4:**
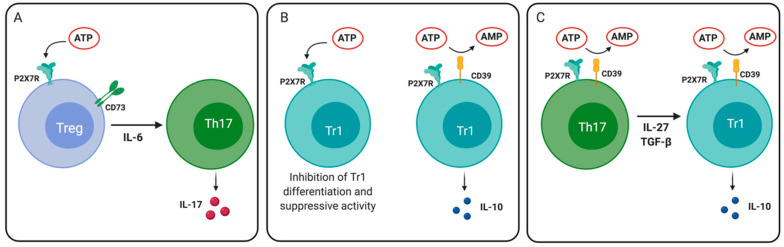
Role of P2X7 receptor in regulatory T cell plasticity and differentiation. (**A**) P2X7R activation in an inflammatory environment (e.g., in the presence of interleukin (IL)-6) can drive the differentiation of Treg cells to Th17 cells. (**B**) ATP signaling through P2X7R reduces the differentiation and IL-10 production by Tr1 cells. CD39 expression on Tr1 cells reduces extracellular levels of ATP, thus allowing IL-10 production. (**C**) Th17 cells that express CD39 may differentiate into IL-10-producing Tr1 cells in the presence of IL-27 and TGF- β1.

In 2016, our group reported further evidence for ATP inhibition of Tr1 responses. In this report, we demonstrated that Th17 cells expressing high levels of CD39 present a plastic phenotype, allowing their differentiation into Tr1 cells in a model of experimental colitis. We reported that Th17 cells differentiated in the presence of TGF- β1 express high levels of CD39 and produce IL-10 (Figure 4). These cells presented an active CD39, since they were less prone to ATP-induced cell death than Th17 cells expressing low levels of CD39. Moreover, IL-10 production and Tr1 differentiation were increased in the absence of P2X7R, suggesting that CD39-mediated ATP depletion allows Th17 cells to differentiate into Tr1 cells [156].

## 5. Concluding Remarks

**Figure 5:**
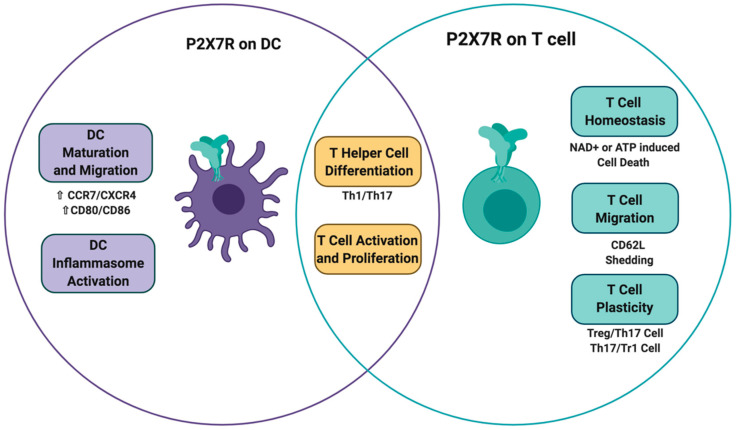
**P2X7 receptor signaling on T cells and dendritic cells impacts T cell fate**. P2X7R activation on dendritic cells triggers DC maturation, migration, and inflammasome assembly. All these effects have an impact on T cell activation and differentiation. On the other hand, the direct activation of P2X7R in T cells can cause different effects that include T cell activation/differentiation, T cell migration, and ultimately T cell death through the induction of ATP and NAD^+^ induced cell death.

The P2X7 receptor is a ligand-gated cation-selective channel whose main physiological ligand is ATP. NAD^+^ may also activate the P2X7 receptor, as it serves as a substrate for ARTC2.2-mediated ADP-ribosylation of this receptor. Several cellular components of the immune system, such as T cells and dendritic cells, express the P2X7 receptor. ATP-mediated P2X7 receptor activation on dendritic cells triggers several responses, such as dendritic cell maturation, migration, and inflammasome assembly, all of which impact T cell activation, promoting the differentiation of inflammatory T helper subsets. On T cells, P2X7 receptor activation may have different outcomes, including the induction of T cell activation/differentiation, T cell migration, and ultimately T cell death through ATP and NAD^+^ induced cell death (Figure 5). Importantly, extracellular ectonucleotidases such as CD39 and CD38 that are present on dendritic cells and T cells, which serve to reduce extracellular concentrations of ATP and NAD, may endow these cells with mechanisms to regulate the extent of P2X7 receptor activation. This NAD^+^ and ATP-induced cell death is context-dependent and may serve to allow the expansion of primed T cells in the lymph node, reduce the overt activation of inflammatory T cell responses in the gut, and allow the expansion of activated resident memory T cells in inflamed tissues. Thus, understanding the delicate regulation of ectonucleotidases and P2X7 receptor expression in different conditions may unravel the impact of purinergic signaling in the adaptive immune response.
